# Investigating the influence of expanded perlite and lime mud on cement-bonded composites containing pulp sludge

**DOI:** 10.1038/s41598-026-39390-x

**Published:** 2026-02-09

**Authors:** Stephen O. Amiandamhen, Carsten Mai, Joran van Blokland, Geoffrey Daniel, Stergios Adamopoulos

**Affiliations:** 1 Department of Wood Technology, Norwegian Institute of Bioeconomy Research, Ås, Norway; 2 Department of Wood Biology and Wood Products, University of Göttingen, Göttingen, Germany; 3 Department of Forest Bioeconomy and Technology, Swedish University of Agricultural Sciences, Uppsala, Sweden

**Keywords:** Cement composites, Sound absorption, Strain analysis, Thermal insulation, X-ray analysis, Engineering, Materials science

## Abstract

This paper presents a comprehensive study on lightweight cement-bonded composites containing pulp sludge (PS). The objective of the study was to evaluate how the incorporation of perlite (a lightweight volcanic glass aggregate) and lime mud (a pulp mill residue) influences composites’ properties including mechanical strength, insulation and fire resistance. Up to 50% of the cement binder was replaced with PS (by mass), and small fractions of cement (5–15%) were replaced with perlite or lime mud. A suite of analytical techniques, material characterization and mechanical tests with digital image correlation (DIC) for strain analysis were employed. X-ray analysis showed that the aggregates influenced the composite properties to a considerable extent due to their particle sizes and ability to form hydrated gels with cement. Adding 5% of perlite or lime mud yields optimal strength without compromising weight reduction whereas higher aggregate content (15%) led to reduced strength. The DIC system provided insights into strain distribution during loading, confirming enhanced toughness from the fibrous PS. The composites were significantly lighter (732–749 kg/m^3^) and showed about 30% lower thermal conductivity (0.17 W/mK) than pure cement composites (0.25 W/mK). The normal incidence sound absorption of the composites was about 0.3 at mid-high frequencies due to their compact structure. The composites demonstrated potential for use as sustainable, lightweight construction materials with good acoustic and thermal insulation, as well as acceptable load-bearing capacity for non-structural applications based on EN 634-1/-2 requirements for cement-bonded particleboards.

## Introduction

Cement-bonded composites are a diverse group of inorganic bonded materials that have different areas of application in civil engineering, construction and repairs, etc.^1,2^. Although the technology of cement-bonded composites is as old as civilization, the rationale for its continuous use lies in the ability to incorporate different materials in the cement matrix, either as reinforcement or fillers to improve the mechanical characteristics and/or environmental footprint of the resulting product^3^. These composites are increasingly incorporating industrial waste materials and lightweight aggregates to improve sustainability and reduce weight. For decades, there has been wide exploration on the use of lignocellulosic materials as reinforcements in the cement matrix, with corresponding flexibility in design of structures with superior properties^4–6^. Some have been developed as thermal insulation materials for building envelope^7–9^. Among the studied lignocellulosic materials with a high potential for availability and performance is pulp sludge from the pulp and paper mill processing residues. Pulp sludge (PS) possesses variable physical and chemical properties that can alter the property of the base material^10^. It is composed mainly of lignocellulosic fibres and inorganic fillers like calcium carbonate^11^. Compared to other natural fibre materials, PS requires less preparation and modification to make it suitable as a cement reinforcement material, and it has also been used as aggregate in cement matrix^12,13^.

To modify certain properties of the composite, aggregates are used in cement as admixtures or replacement materials. Researchers have demonstrated that different materials used as aggregates can improve the formation of silica hydrates, chloride-ion penetration resistance, drying shrinkage microcracking, insulation properties, and enhance mechanical properties of the cement composites^14–17^. This study investigates two types of aggregates viz lime mud (LM) and perlite (PL). LM is a pulp and paper residue that originates from the causticization process during chemical recovery. LM is fine and chemically like limestone, making it a candidate for partial replacement of cement or lime in construction materials. Due to its high alkalinity and mineralogical properties, it is regarded as a toxic industrial waste that should be treated before discharge^18^. PL is a white siliceous volcanic glass that contains combined water within its structure. Its unique characteristics include low density, chemical inertness, particle structure, liquid absorbency, high specific surface, and 20 × volume expansion when heated^19,20^. Whereas LM has been studied as a filler for many years, expanded PL is a newer pozzolanic aggregate in concrete research. Due to its low bulk density, it is commonly used as insulating or lightweight filler in concrete^21^. Both materials yield lightweight, good thermal insulation properties, and reduced strength to building structures when used as cement aggregates. However, thermal insulation materials must also have good sound absorption, acceptable strength, and adaptability^22^.

The aim of this study therefore was to investigate the influence of PL and LM as cement replacement aggregates on the mechanical strength, thermal, acoustic and fire resistance properties of pulp sludge cement composites. The broader aim was to develop a lightweight, insulation cement composite material using waste by-products without compromising performance. In a previous study, Borinaga-Trevino et al.^23^ showed that up to 20% of cement could be replaced by LM without any significant effect on the thermal and mechanical properties of the composite. Rizqian et al.^24^ also observed increased compressive strength in mortars at 28 days with an optimum lime content of 5% but with an additional 2% superplasticizer, reaching 16.29 MPa compared to ordinary mortar of 4.74 MPa. However, Patthanavarit et al.^25^ found decreased flexural strength when PL content increased to 15% in cement mortar, with values ranging between 7.8 and 12.5 MPa. Jaworska et al.^26^ also reported a reduction in both tensile and compressive strength properties of composites incorporating waste perlite (4% and 2%, respectively) after 28 days. These studies separately evaluated LM as cement filler (often at higher replacement levels) and PL for lightweight mortars. However, a unified, multifunctional assessment combining PS with LM or PL at low aggregate dosages and mapped by full-field DIC has been lacking. The existing studies rarely optimize C/PS ratios simultaneously with LM/PL fractions for multi-property performance and quantify strain localization and crack paths in PS-cement systems via DIC. This study addresses these gaps by: (a) using PS as a base filler (up to 50 wt.%) with small LM/PL additions (5–15 wt.%); (b) optimizing C/PS at a fixed 5 wt.% aggregate; (c) performing full-field DIC during compression test; and (d) systematically evaluating flexural, compressive, acoustic, thermal and fire reaction properties.

## Materials

**Fig. 1:**
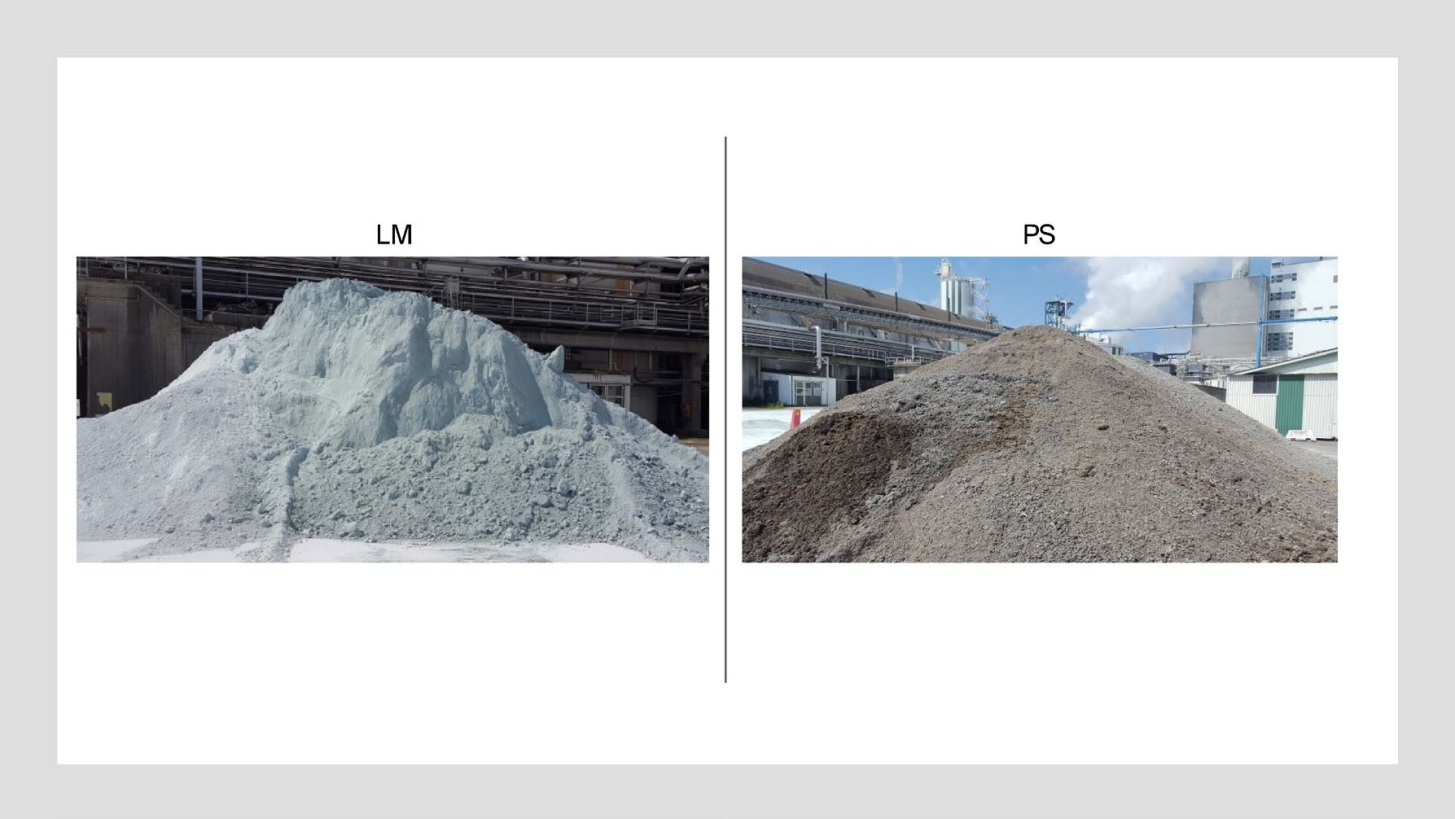
Pulp mills’ residual materials; lime mud (LM) and pulp sludge (PS) (*Source*: Photos taken at Södra Cell Värö, Sweden).

The materials used in the study included pulp sludge (PS), which was collected from Södra Cell Värö, Väröbacka, Sweden. The PS (55.1% moisture content) was dewatered and dried before use, leaving a fibrous, fine particle mix that can act as a fibre reinforcement and filler. The aggregates used were lime mud (LM) and perlite (PL). LM was also obtained from Södra Cell as residuals, while PL was supplied by Scandinaviska IFAB filtrering AB, Torslanda, Sweden. The pulp mill residual materials are pictured in Fig. 1. The LM was an off-white, powdery material and was used in its as-received wet state (35.4% moisture content). The PL used was an expanded and low-density powdery material obtained from calcination of lava sand at 1100 °C. It consists mainly as diatomite, a near pure sedimentary silica deposit. The cement used was ASTM type II Ordinary Portland Cement (CEM II/A-LL, 42.5 R). The particle size distribution and chemical composition of the materials are presented in Tables 2 and 3.

## Methods

### Mix design and composite production

The wet PS was air-dried at ambient conditions to prevent the growth of mold. The moisture content of the air-dried PS was 6.2% at the time of use. The mix design of the composites is presented in Table 1. The PS was premixed for 10 min with 100 wt.% water in a mechanical mixer (Hobart HSM 30-F3E, Peterborough, UK) to attain the saturated surface dry (SSD) condition based on preliminary trials. Thereafter, cement, aggregates and water were measured and added to the mix. The water to cement (w/c) ratio was kept constant at 0.6. This w/c ratio provided stable workability under SSD for PS. Higher w/c increased segregation whereas lower w/c impaired dispersion given PS absorbency. The absorptivity of LM and PL was accounted for via SSD conditioning, keeping total free water at 0.60 to ensure reproducibility. The mixture was agitated for 5 min, and the composites were formed in wooden molds measuring 350 × 270 × 50 mm^3^ (for flexural tests) and 160 × 150 × 50 mm^3^ (for compression tests). The composites were then pressed at room temperature with an applied pressure of 2 MPa for 10 min. The applied pressure balances de-airing, fibre alignment minimization and thickness control without excessive water squeeze-out. The target thickness of the composites was 12 mm and 25 mm for flexural and compression tests, respectively. The formed composites were air-cured at 20 °C and 70% relative humidity (RH) for 28 days (standard for cement hydration with lignocellulosic materials). Samples were kept on spacers to promote uniform drying and mitigate thickness-driven moisture gradients. Initially, the effect of aggregate content (5, 10, 15 wt.%) was investigated on the bending strength properties. Thereafter, the cement to PS ratio was optimized according to Table 1. Several rationales were considered for the selected ranges in both phases of the mixture design. Low aggregate fractions (5–15 wt.%) are widely reported to balance mechanical response and insulation. Our preliminary trials indicated diminishing strength beyond 15%, hence the chosen bounds. Further, PS at 50% targets high waste utilization while retaining adequate cohesion and workable rheology per prior PS-cement studies. Finally, the C/PS identifies a practical trade-off between stiffness, strength and insulation for panel design.

**Table 1:** Mix design for the PS composites.

Binder (cement + aggregate)	PS	W/C
Investigating the effect of LM/PL (wt.%)		0.6
50% cement (control)	50% PS	
45% cement + 5% LM/PL	50% PS	
40% cement + 10% LM/PL	50% PS	
35% cement + 15% LM/PL	50% PS	
Optimizing cement to PS ratio (w/w)		0.65
70% cement (control)	30% PS	
65% cement + 5% LM/PL	30% PS	
55% cement + 5% LM/PL	40% PS	
45% cement + 5% LM/PL	50% PS	
35% cement + 5% LM/PL	60% PS	

### Characterization

The particle size (length, thickness density) distribution of the cement, LM and PL was carried out using FibreShape PRO (X-shape, IST, Vilters, Switzerland). Samples were oven-dried (105 °C to constant mass), gently disaggregated and dispersed in a transparent film such that they do not overlap. High resolution images were captured using a flatbed scanner (Epson Perfection V850 Pro, Epson, Tokyo, Japan) in transmitted light mode. The scans were loaded to the FibreShape software, and the particle size was assessed by static image analysis. X-ray fluorescence (XRF) spectrometer (Malvern Panalytical, Malvern, UK) was used to analyze the composition of the cement and aggregates (LM and PL). X-ray diffraction (XRD) (Malvern Panalytical Empyrean, Almelo, the Netherlands) diffractometer equipped with a Cu LFF HR X-ray tube, programmable anti-scatter slit, and a PIXcel3D detector was employed to determine the phase of the samples. The scanning degrees in 2θ range was 5 to 60° with a scanning speed of 1°/min at 45 kV and 40 mA. The samples were milled using a mixer mill (MM 400, Retsch, Germany) before X-ray analyses. The microstructure of the fractured surface of the samples was observed using a XL30 environmental scanning electron microscopy (ESEM) (Thermo-Fisher, Eindhoven, the Netherlands) with secondary electrons in conventional mode. The characterization was performed at an accelerating voltage of 10—20 kV and a working distance of 10 mm. Specimens were mounted on pin stubs and sprayed with gold using a high vacuum EMITECH K550X sputter coater prior to imaging.

### Drying rate

The drying rate of the samples was measured over a curing period of 28 days at 7 days interval based on their densities. The density of the composites was measured based on their dry weight and volume at ambient conditions as outlined in EN 323^27^ using samples measuring 50 × 50 × 12 mm^3^.

### Mechanical tests

Mechanical tests were conducted using an MTS Exceed Test System (MTS Systems Norden AB, Askim, Sweden) fitted with a 10 kN load cell. Three-point flexural tests were carried out according to EN 310^28^ at a crosshead speed of 3 mm/min using 290 × 50 × 12 mm^3^ samples. Modulus of rupture (MOR) and apparent modulus of elasticity (MOE) were derived after the test. Compression tests were conducted according to ASTM D1037^29^ using 100 × 25 × 25 mm^3^ samples at a crosshead speed of 1.5 mm/min. The measurements were performed in four replicates, and the mean values were plotted with standard deviations. The dimensions reflect standard fixtures and avoid slenderness artefacts; flexural thickness set to 12 mm to target panel applications; compression thickness 25 mm allows DIC-compatible speckle resolution.

### ARAMIS digital image correlation (DIC)

An ARAMIS DIC system was used to analyze surface strain and crack propagation in the composite samples during compression tests. Samples measuring 75 × 25 × 25 mm^3^ were prepared following ASTM D1037^29^ compression test for short columns. One side of the samples was prepared with a black on white speckled pattern. One test per sample was performed and only one side of the column was monitored. In total, nine samples were analyzed. The relative displacement on the surface was recorded by a non-contact optical deformation measurement system (Aramis adjustable, GOM GmbH, Germany). The loading force, crosshead displacement and time for the compression test were imported into the Aramis system. The device employs two cameras with 75 mm lenses at 450 mm distance from the object, each with 4096 × 3072 pixels full frame resolution. The measuring volume was 120 × 90 × 70 mm^3^ (width, height and depth). The strain reference length was 0.8 mm; facet size was 25 × 25 pixels and point distance was 15 pixels. These were selected to satisfy the recommended ratio of speckle size ≥ 3–5 pixels and ensure sub-pixel correlation stability while resolving crack-scale strain bands; the 0.8 mm reference length balances noise and spatial resolution. Frames were collected from the start until the end of test at a rate of 1 frame per second. As the applied load increases and the sample deforms, the DIC software (GOM Correlate, GOM) computes full-field displacements and strains by comparing images frame by frame. Alignment was checked via pre-load frames, platen parallelism was adjusted, eccentricity was flagged and excluded from strength statistics where relevant.

### Acoustic properties

The acoustic properties of the composite samples were performed according to ISO 10534-2^30^. The normal-incidence complex acoustic impedance and sound absorption coefficient were measured using the ACUPRO system (TFAcoustics, LLC, USA). Samples measuring 34 mm in diameter and 25 mm thick were used and the test was performed in three replicates. Because 25 mm sample thickness may reduce sensitivity in < 200–300 Hz range, interpretations focus on mid-high bands.

### Thermal analysis

The thermal conductivity of the composite samples was measured using the transient plane source (TPS) method according to ISO 22007-2^31^. The test was conducted on the assumption that the samples are isotropic and homogenous. The samples were selected based on the different PS to cement ratio (as shown in Table 1). Control samples were also measured to investigate the effect of the aggregates on thermal properties. 28-days cured samples were cut into dimensions of 50 × 50 × 25 mm^3^ and were conditioned at 20 °C and 50% RH to constant weights and 6% moisture before the test. The samples were paired as couples, with each sample having two surfaces (S_1_ and S_2_). Measurements were performed for a combination of surfaces in the same sample couple.

### Cone calorimetry test

The heat release rate (HRR), total heat release (THR) and time to ignition (TTI) were evaluated using a mass loss calorimeter (MLC, Fire Testing Technology, East Grinstead, UK) following ISO 5660-1^32^. Tetragonal specimens (100 × 100 × 25 mm^3^) were exposed to heat flux of 50 kW/m^2^ for 30 min. The measurements were performed in three replicates, and the mean values were plotted.

### Data analysis

The experiment was laid out in a completely randomized design using the Minitab statistical software (Minitab, LLC, Pennsylvania, USA). A one-way analysis of variance procedure was conducted to analyze the effect of the PS types and C/PS ratio on the composites’ properties at 5% level of significance. Tukey’s honestly significant difference test was used for pairwise comparison of means at α = 0.05.

## Results and discussions

### Materials and composite characterization

#### Particle size distribution and elemental composition

The particle size distribution of cement and aggregates is presented in Table 2, and their chemical composition is presented in Table 3. The particle size analysis confirmed that LM is an ultrafine material (d_50_ ~ 27 µm) compared to PL (d_50_ ~ 34 µm) and cement (d_50_ ~ 44 µm). PL has a bimodal size profile with some coarse particles (see Fig. 3). Fine particle additives improve packing by filling voids but require careful water adjustment^33^. In this study, a well-graded mix was achieved by combining these components, which benefitted the packing density. CaO and SiO_2_ are the main components that affect hydration and pozzolanic reactions, which influence strength development in cement composite materials^34^. Table 3 shows that the incorporation of LM increases the overall CaCO_3_ content in the cement mix (via a high CaO in the ash analysis). The implication of this is an increased dilution of cement’s clinker phases and the potential reaction with any free portlandite Ca (OH)_2_ from cement hydration^35^. Fine LM particles likely fill in the spaces between cement grains, providing a micro-filler effect and potential nucleation sites for C-S–H during hydration^36^. This could be responsible for the slightly higher mechanical properties in samples containing LM compared to those of PL. High packing density could have also contributed to the significant difference in dry densities between both composite types. PL contains a high amount of silica, which is an important pozzolanic material for producing hydrates^37^. Although both aggregates have distinctive mineralogical composition, they influence the composite properties to a considerable extent due to their particle sizes and ability to form hydrated gels with cement.

**Table 2:** Particle size distribution of cement and aggregates.

Density (kg/m^3^)		Particle size (µm)						
		d_0_	d_5_	d_10_	d_50_	d_90_	d_95_	d_100_
CEM II	1250	14.11	19.39	21.04	44.38	108.63	140.38	848.55
LM		14.11	14.87	14.87	28.61	77.86	104.44	196.99
PL	80	10.52	14.87	14.87	33.92	87.49	122.16	494.25

Values represent the particle diameter at 0–100 percentiles (d_0_–d_100_).

**Table 3:** Chemical composition of cement and aggregates.

	Chemical composition (wt.%)											
	CaO	MgO	SiO_2_	Al_2_O_3_	MnO	Na_2_O	K_2_O	P_2_O_5_	SO_3_	Fe_2_O_3_	BaO	Cl
CEM II	64.2	1.12	18.6	5.19	0.48	0.05	1.7	0.12	5.29	2.87	0.06	0.02
LM	54.2	1.4	0.14	0.05	0.07	1.07	–	0.94	0.08	0.03	–	0.03
PL	0.77	0.11	75.8	13.8	0.05	2.84	5.75	< 0.01	–	0.6	0.13	0.04

#### X-ray diffraction (XRD)

**Fig. 2:**
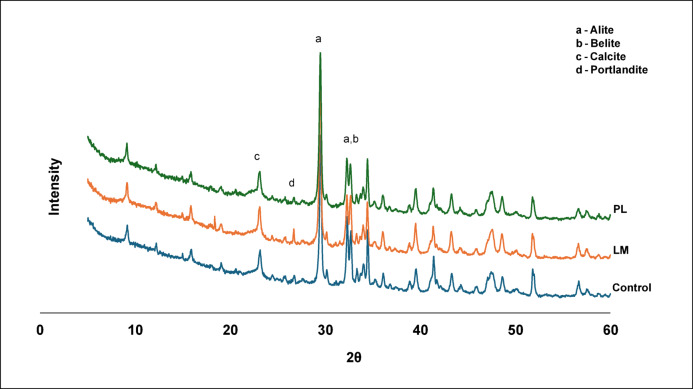
XRD spectra of the PS composites.

The XRD spectra (Fig. 2) of hydrated samples did not show any new crystalline calcium silicate hydrates (C-S–H). However, the portlandite content in composite containing PL was slightly reduced compared to the LM composite and control (based on the relative intensity of Ca (OH)₂ peak). This suggests that there was some consumption of Ca (OH)₂ most likely by pozzolanic reaction with the high amount of amorphous silica in PL or the siliceous components of PS^38^. The absence of crystalline pozzolanic products peaks (i.e., no clear C-S–H) implied that the reaction was limited or the products remained mostly amorphous. The presence of calcite (CaCO_3_) in the LM sample was higher, probably due to unreacted LM or carbonation. It is also important to know that there were no deleterious phases (e.g., expansive ettringite) observed. This could be attributed to the very low content of sulphate in LM and PL introduced no sulphate (Table 3). Thus, chemically, the composites appear stable. LM shows a higher intensity of alite (C_3_S) and belite (C₂S) due to its calcium content. The high CaCO_3_ from LM can help to refine pore structure and improve strength by filler packing and nucleation without chemical reaction^39^. This also explains the higher strength values obtained in LM composites compared to PL.

#### Scanning electron microscopy (SEM)

**Fig. 3:**
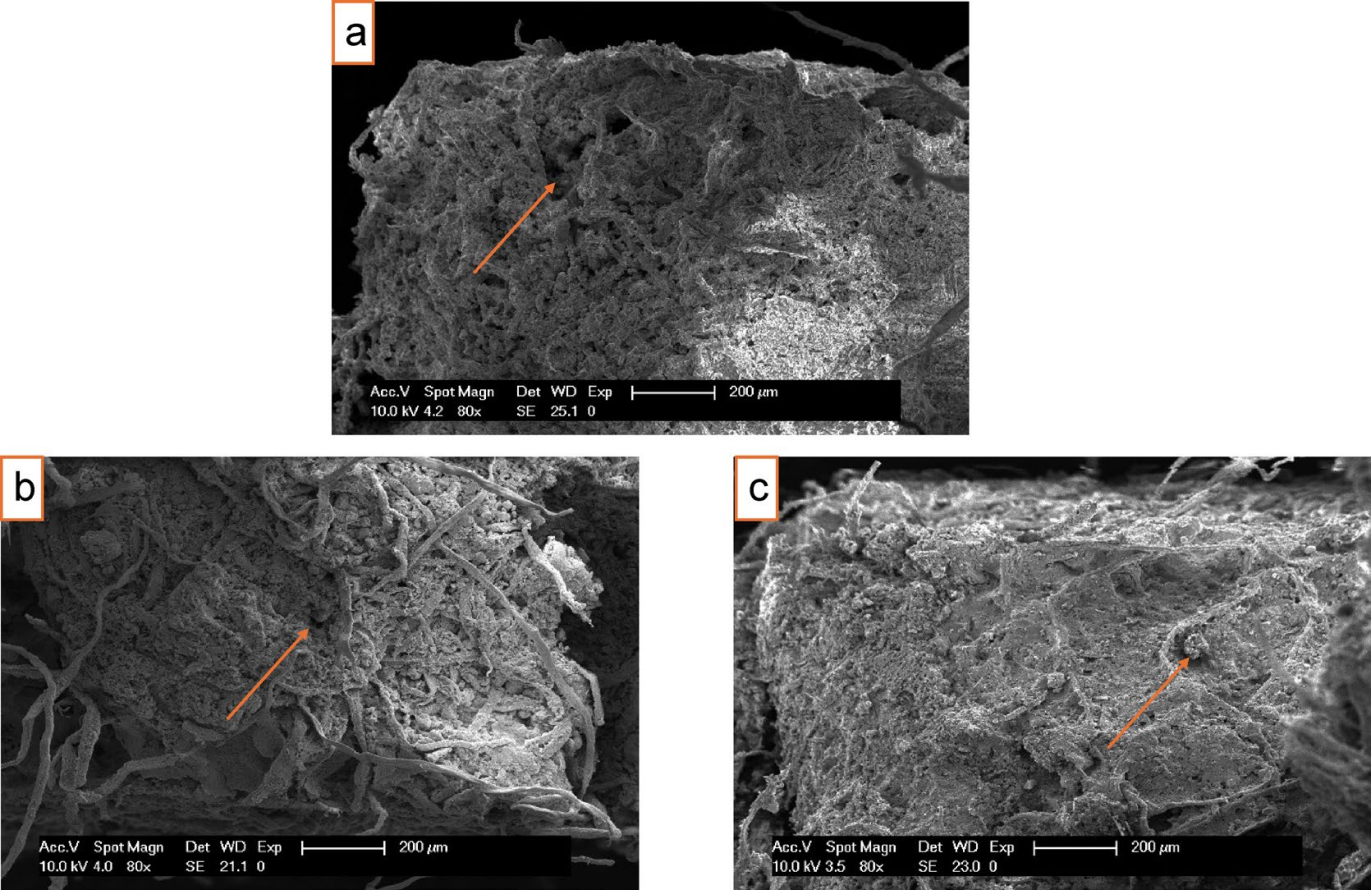
SEM micrographs of fractured surfaces; (**a**) Control showing porous matrix and some fibre pull-out, (**b**) Composite with LM showing denser matrix with fewer large voids, fibres still evident, (**c**) Composite with PL showing an expanded particle and a generally compact matrix with fibre bridging.

**Fig. 4:**
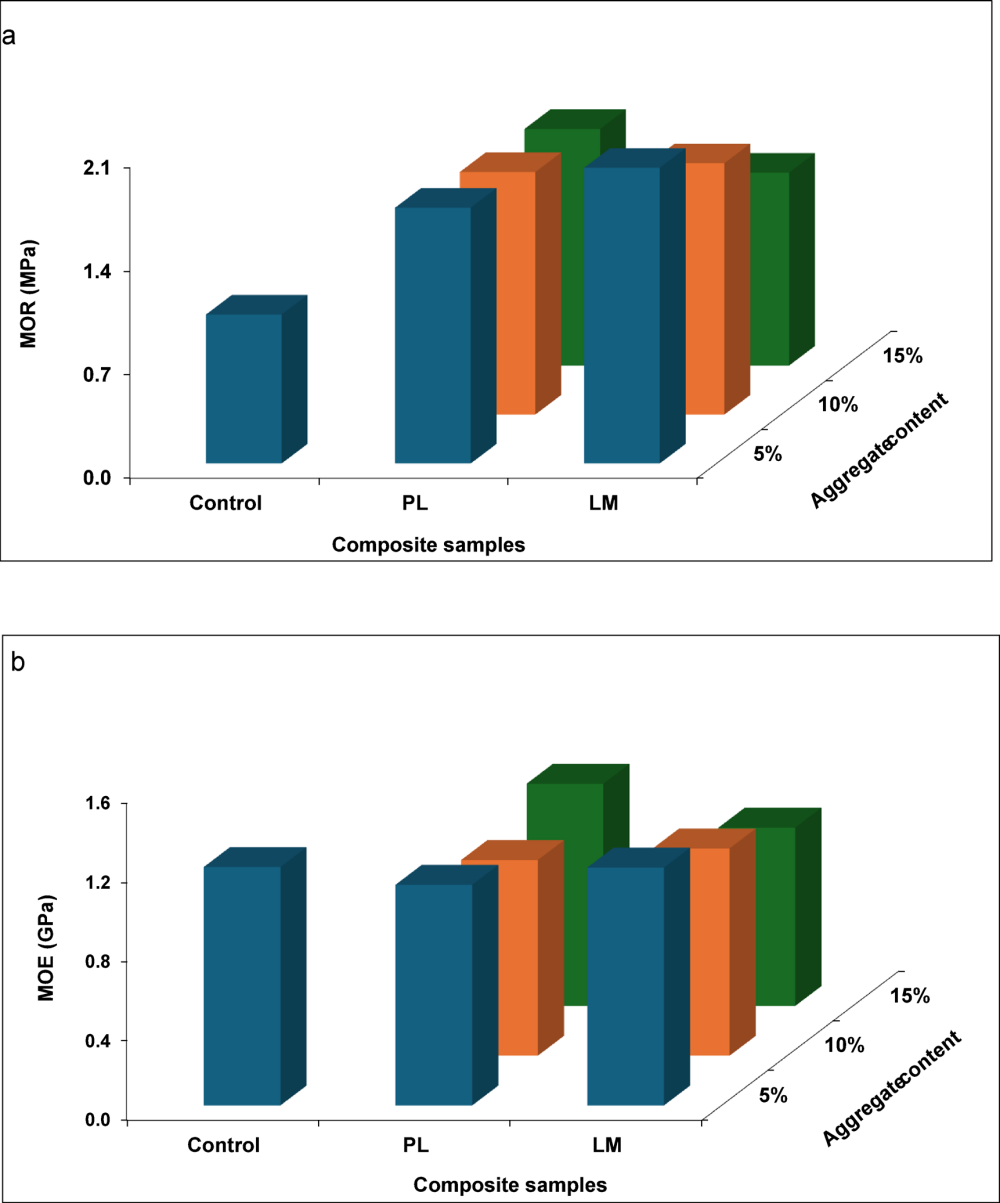
Flexural strength of 28-days cured samples at different aggregate content; (**a**) MOR (**b**) MOE.

SEM imaging (Fig. 3) revealed a porous and fibrous reinforced microstructure of the composites. At equal degree of magnification (200 µm), the PL composites show more dense packing compared to those containing LM. The expanded particles of PL appeared largely intact without much sign of disintegration in the cement paste, confirming their inert role (Fig. 3c). More micropores could be observed at the interface of LM composites, but it generally appeared good with sufficient hydration products (Fig. 3b). PS fibres could be observed bridging cracks and pores, with most fibres remaining embedded in the matrix. However, a few pulled-out fibres were also observed. These fibres are likely to enhance post-crack load capacity in the composites^40^. The micrographs of the aggregate-filled samples (Fig. 3b, c) also showed clusters of fine particles filling smaller voids, which were absent in the control sample (Fig. 3a). This micro-filler effect could explain the increase in 28-day strength observed in composites with PL and LM compared to those of the control (Fig. 4a). Fine particles of the aggregates could densify the microstructure by occupying spaces between cement grains resulting in some marginal increase in compressive strength^41^. A similar increase was also observed in this study. Overall, SEM observations confirmed the intended roles of PS and the aggregates in the composites, which was to provide crack bridging networks and fill micropores, respectively.

### Mechanical properties

#### Modulus of rupture (MOR) and apparent modulus of elasticity (MOE)

**Fig. 5:**
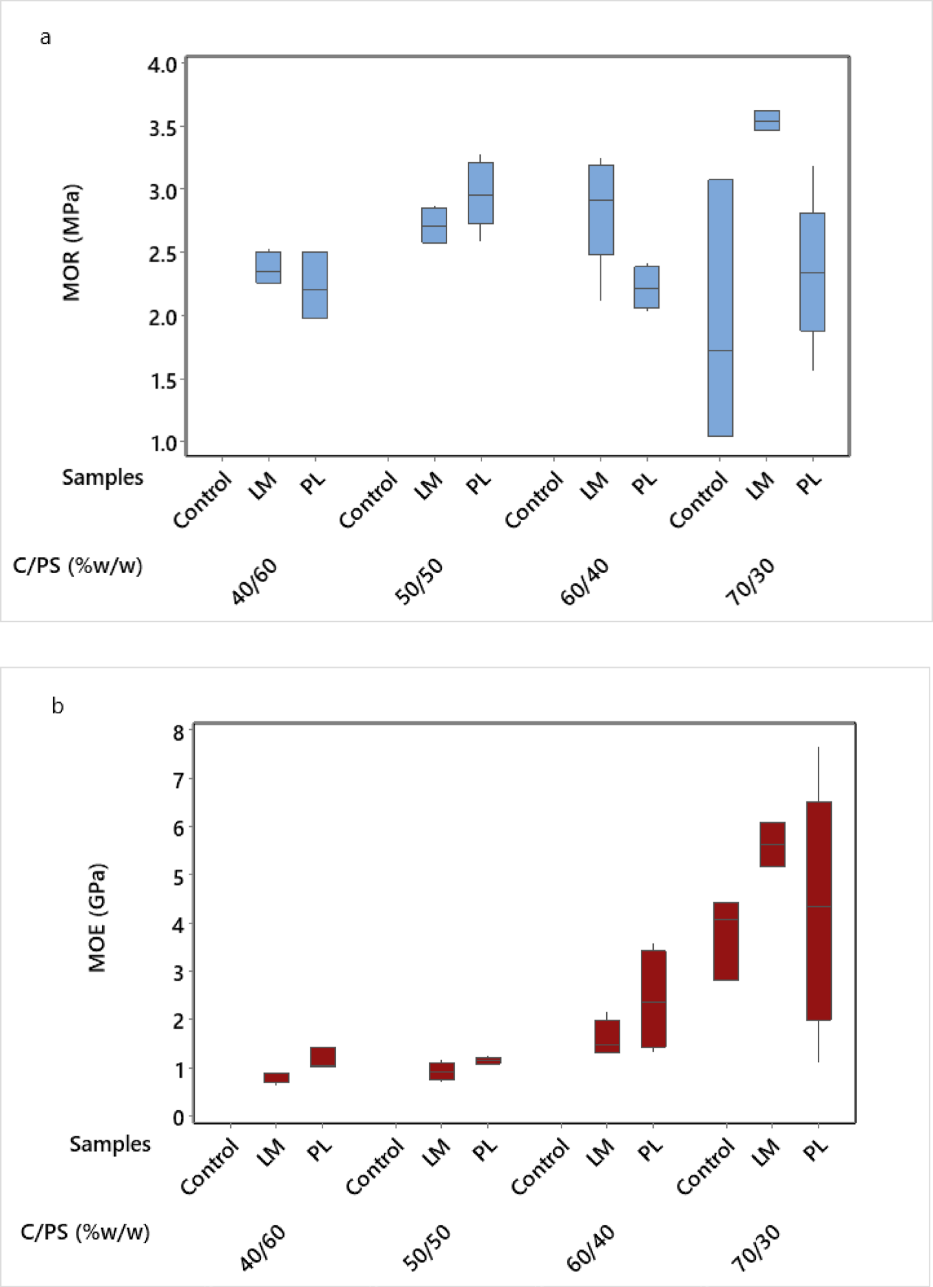
Flexural strength of 28-days cured samples at different C/PS ratios: (**a**) MOR (**b**) MOE.

The initial flexural properties (MOR and MOE) results of 28-days cured samples are presented in Fig. 4a, b. The first step was to investigate the effect of the aggregates at different contents (wt.%) on the evaluated properties. The composites were made at equal proportion (w/w) of PS and binder (cement + aggregate), i.e., 50:50. Subsequently, the results were used to optimize binder to PS ratio (C/PS, w/w) according to Table 1. Based on the initial results, the final composites were made with 5% aggregate content and the C/PS ratio varied in the optimization process. The results are presented in Fig. 5a, b. PS samples containing the aggregates had a higher mean value in MOR compared to the control samples at all levels of the aggregate. LM samples also had higher MOR than PL samples. A similar pattern was observed for the MOE, although the control sample had a higher mean value in MOE than those containing aggregates. MOR decreased with increasing aggregate content, but the difference was not significant. Jaworska et al.^42^ reported a 65% decrease in flexural strength in cement composites with a 6 × increase in waste PL. The strength improvement effect of LM can be further enhanced with additives such as silica fume, and their combined effect in mortar can improve flexural strength by about 60%^43^. In the optimization phase (Fig. 5), the highest MOR and MOE values (3.55 MPa and 5.63 GPa, respectively) were recorded for a C/PS ratio of 70/30. This range aligns with wood-cement panels for lightweight boards^2,44^. Notably, higher cement content increases the flexural properties. C/PS ratio showed a significant effect on the MOE but the effect on the MOR was not significant. On the contrary, the composite type had a significant effect on the MOR but its effect on the MOE was not significant (α < 0.05).

#### Compressive strength (CS)

**Fig. 6:**
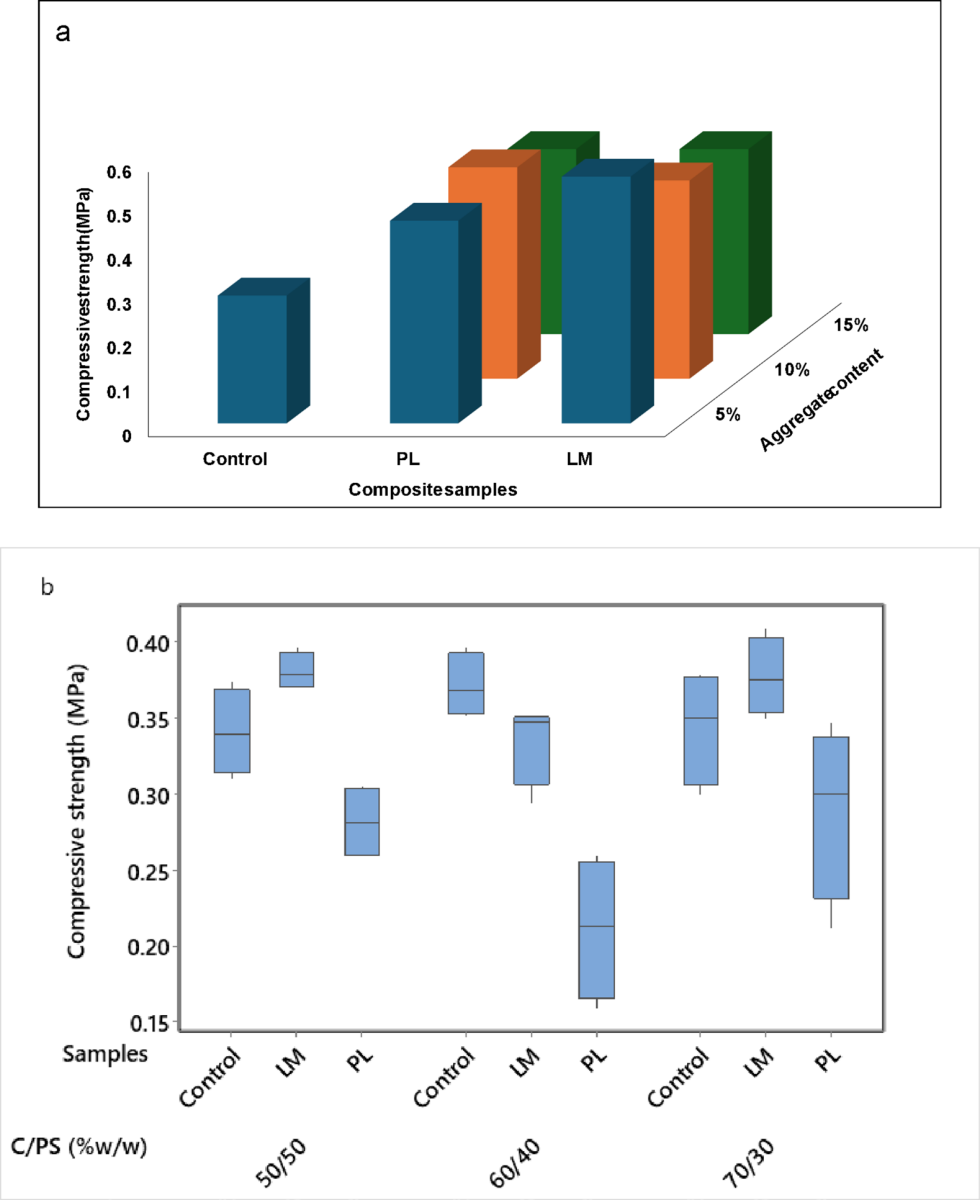
Compressive strength of 28-days cured PS composites: (**a**) Investigating aggregate content, (**b**) Investigating C/PS ratios.

A similar pattern of development was observed in the compressive strength (CS) of the PS composites (Fig. 6). The CS increased at all levels of aggregates compared to the control. The control composite had a mean CS of 0.29 MPa at 28 days. Adding 5% PL or LM increased this to 0.46 and 0.42 MPa (representing about 40 and 48%), respectively (Fig. 6a). At 15% LM content, the CS decreased by about 25% while that for PL was just 8.7%. The decrease could largely be due to the proportionately reduced cement content. These values are suitable for non-structural panel applications where low density and insulation dominates. Like the flexural properties, the result was used to optimize the C/PS ratios. The incorporation of LM yields higher CS than PL at all levels of C/PS (Fig. 6b). The presence of CaCO_3_ in LM can significantly influence hydration kinetics by providing nucleation sites, thereby accelerating early-age reaction and increased compressive strength^45^. Contrarily, the CS of PL composites decrease for all levels of C/PS. Other authors also reported decreased CS in cement composites containing PL^25,42^. This likely suggests that the increased porosity caused by PL has a detrimental effect on the load-bearing capacity within the material. The composite type had a significant effect on the CS (although there was no mean difference between LM and control composites), whereas the effect of the C/PS ratio was not significant (ɑ < 0.05). The results of the optimization were used to further develop PS composites for the DIC evaluation.

### Digital image correlation (DIC)

**Fig. 7:**
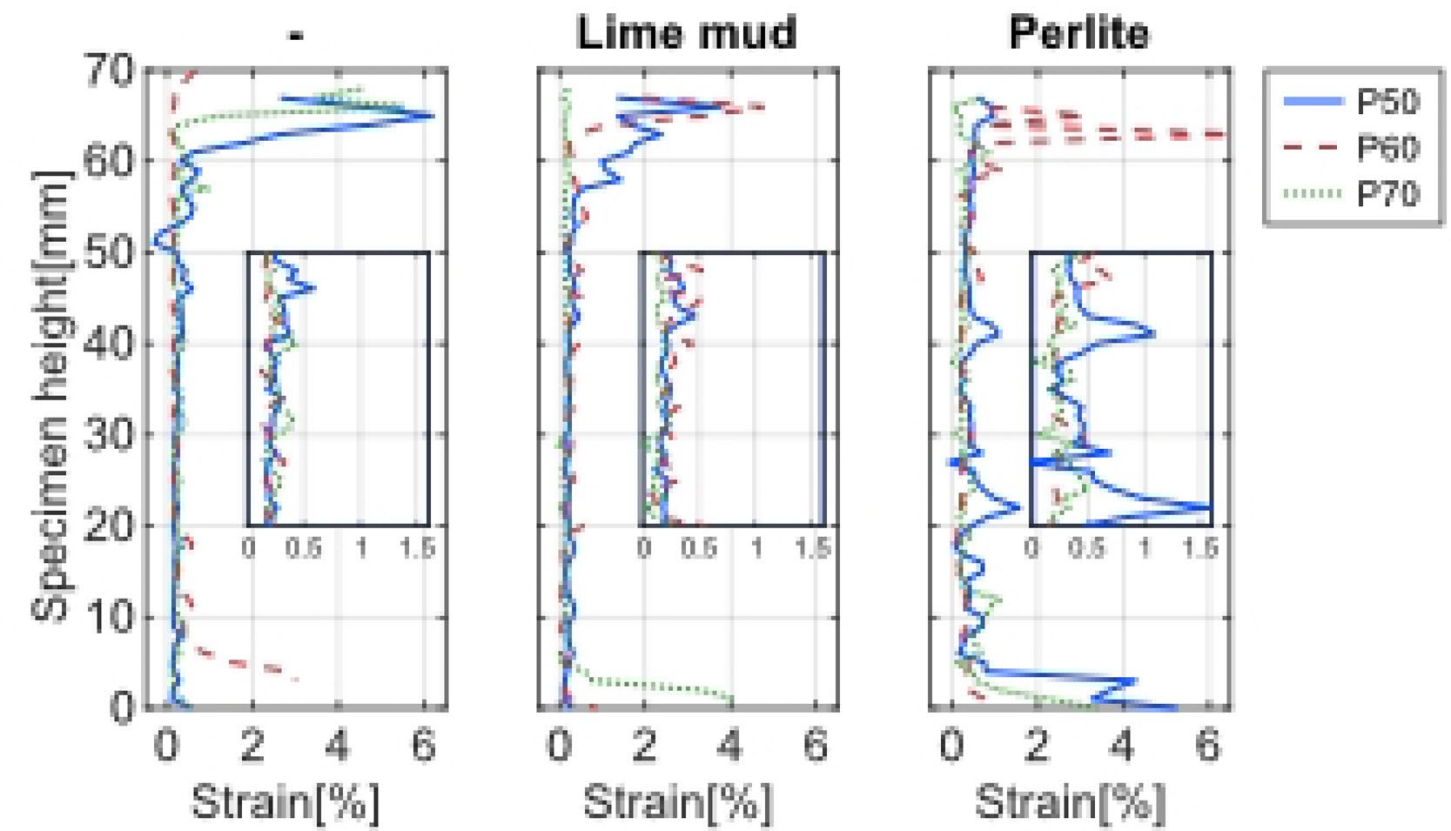
Average compressive strain (*ε*_x_) plotted over sample height when loaded (*σ* = 0.2 MPa). C/PS: P50 = 50/50; P60 = 60/40; P70 = 70/30.

**Fig. 8:**
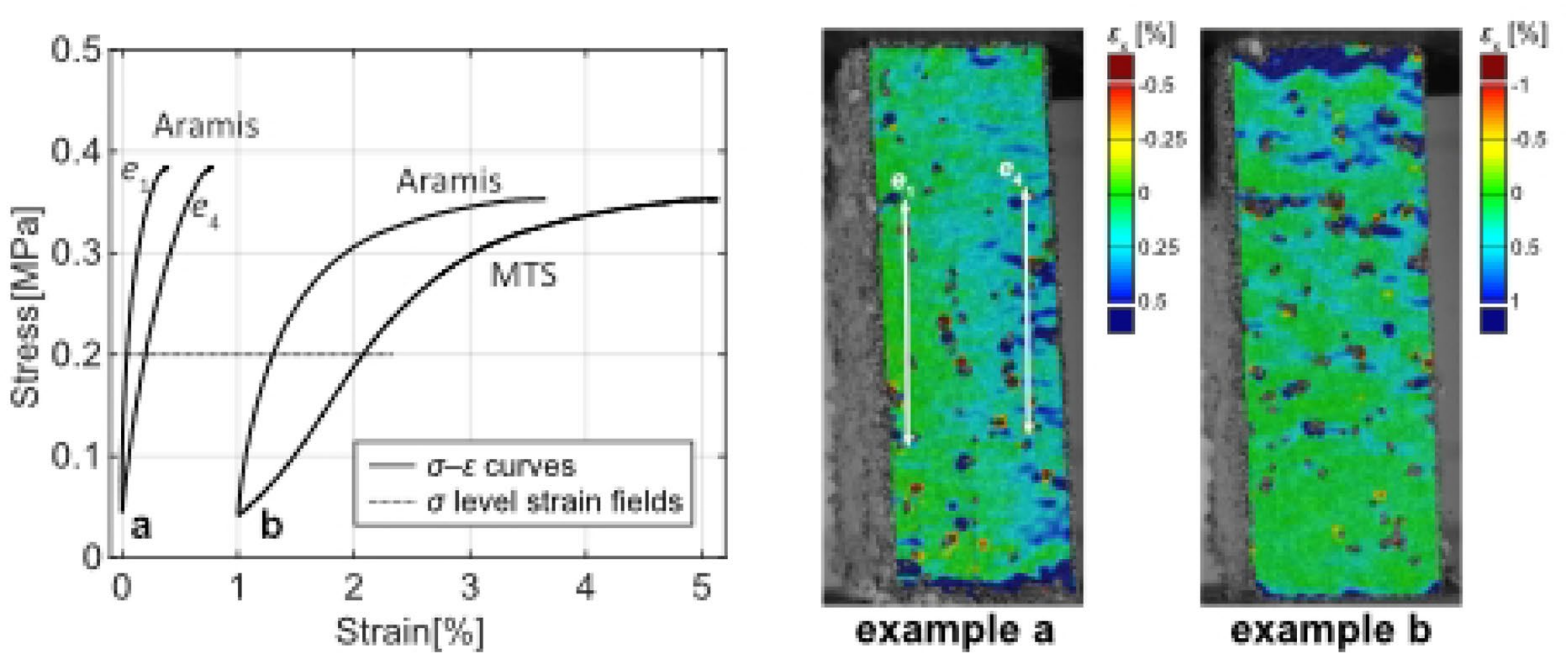
*σ*–*ε* curves and strain fields (*ε*_x_) showing non-uniform loading of samples; (**a**) Eccentricity, (**b**) Local deformation close to loading plate.

**Fig. 9:**
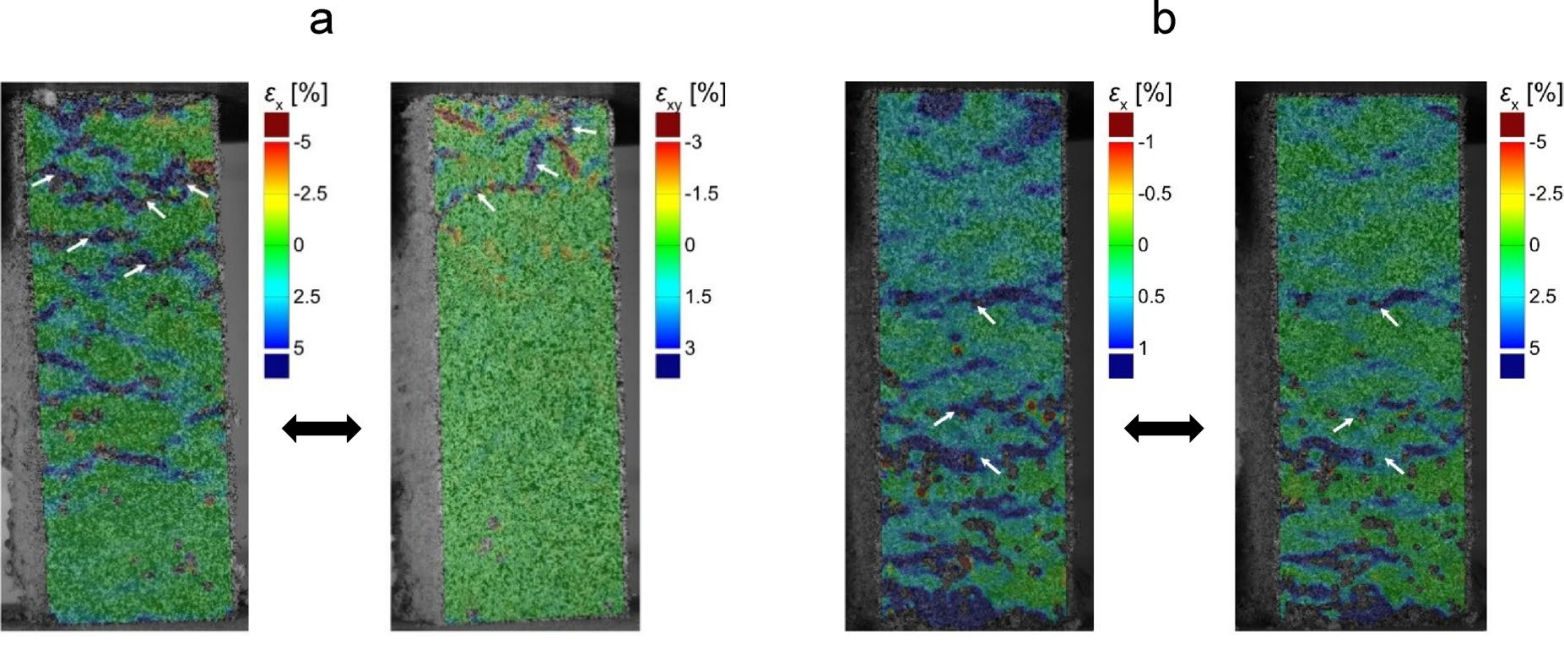
Failure modes during compression tests; (**a**) Compressive strain ↔ Shear strain, (**b**) Compressive strain (*ε*_x_) in PL composite with C/PS of 50/50 (*σ* = 0.2 MPa ↔ 0.3 MPa). Arrows point out failure line (s).

The DIC strain map shows the development of heterogenous strain in the composite samples (Fig. 7). Initially, the strain was uniform, but as load increased, certain regions exhibited localised higher strain. Local strain accumulation suggests inhomogeneous deformation and possibly weak zones. This is often attributed to weak points in the microstructure where microcracks nucleated^46^. This could be for example at the interface around a large PL, LM particle or cluster of pores. As the load reaches the peak and the samples began to fail, vertical splitting cracks propagated. The PS fibres across cracks may have provided some bridging effect. Eventually, a network of cracks formed, and failure occurred by pieces dislodging. Eccentricity (bending moment) was observed in three of the samples (Fig. 8), which could indicate that the compressive strength was underestimated in those cases. Samples showing eccentricity were flagged and the strength data excludes those tests. However, DIC was still retained for qualitative failure-mode mapping. In other samples, final failure may be due to shear close to the supports (Fig. 9). This also could mean that the results did not represent compressive strength but shear strength of the material. Compared to pure mortar, the composite exhibited more distributed strain fields. The influence of PS in bridging cracks led to a more diffuse damage pattern.

### Drying rate

**Fig. 10:**
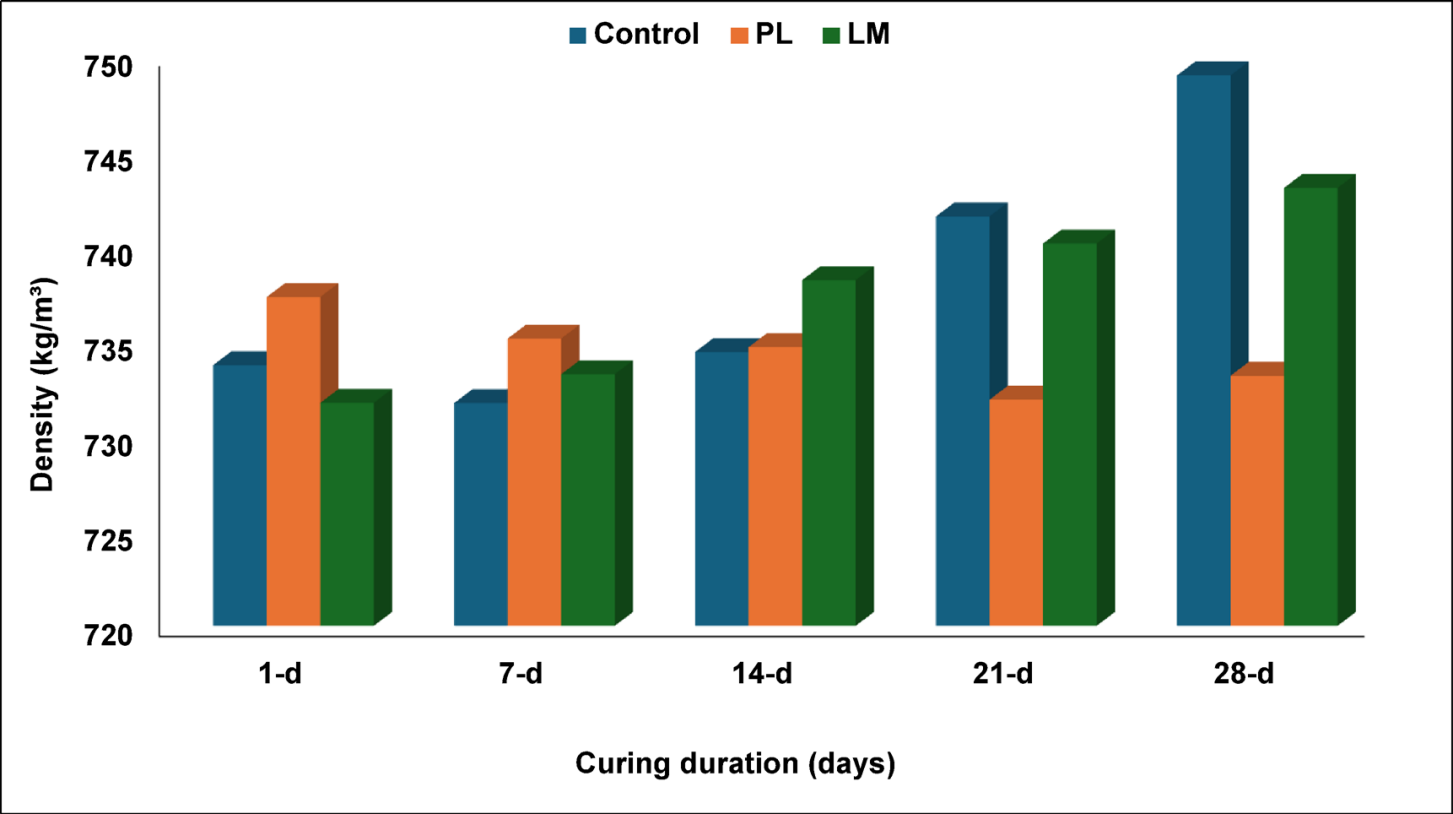
Drying rate of the PS composites.

The drying rate of the PS composites shows the development of density as hydration progresses (Fig. 10). Whereas density increased for both control and composites containing LM, density decreased slightly in composites containing PL as curing progresses to 28 days. The mean density ranged from 734 to 749 kg/m^3^ for the control composites, 732–743 kg/m^3^ for the LM composites and 737–733 kg/m^3^ for the PL composites. As a concrete material dries, the water content decreases, resulting in the formation of a denser, more compact material^47^. The slight decrease observed in PL composites may be attributed to the porosity of the material. As the composite cures and dries, the internal voids within the PL particles and between the particles may not fully consolidate, leading to a slightly lower overall density. Additionally, if the drying process leads to significant water loss without substantial volume reduction, the density of the composite will decrease^48^.

### Acoustic properties

**Fig. 11:**
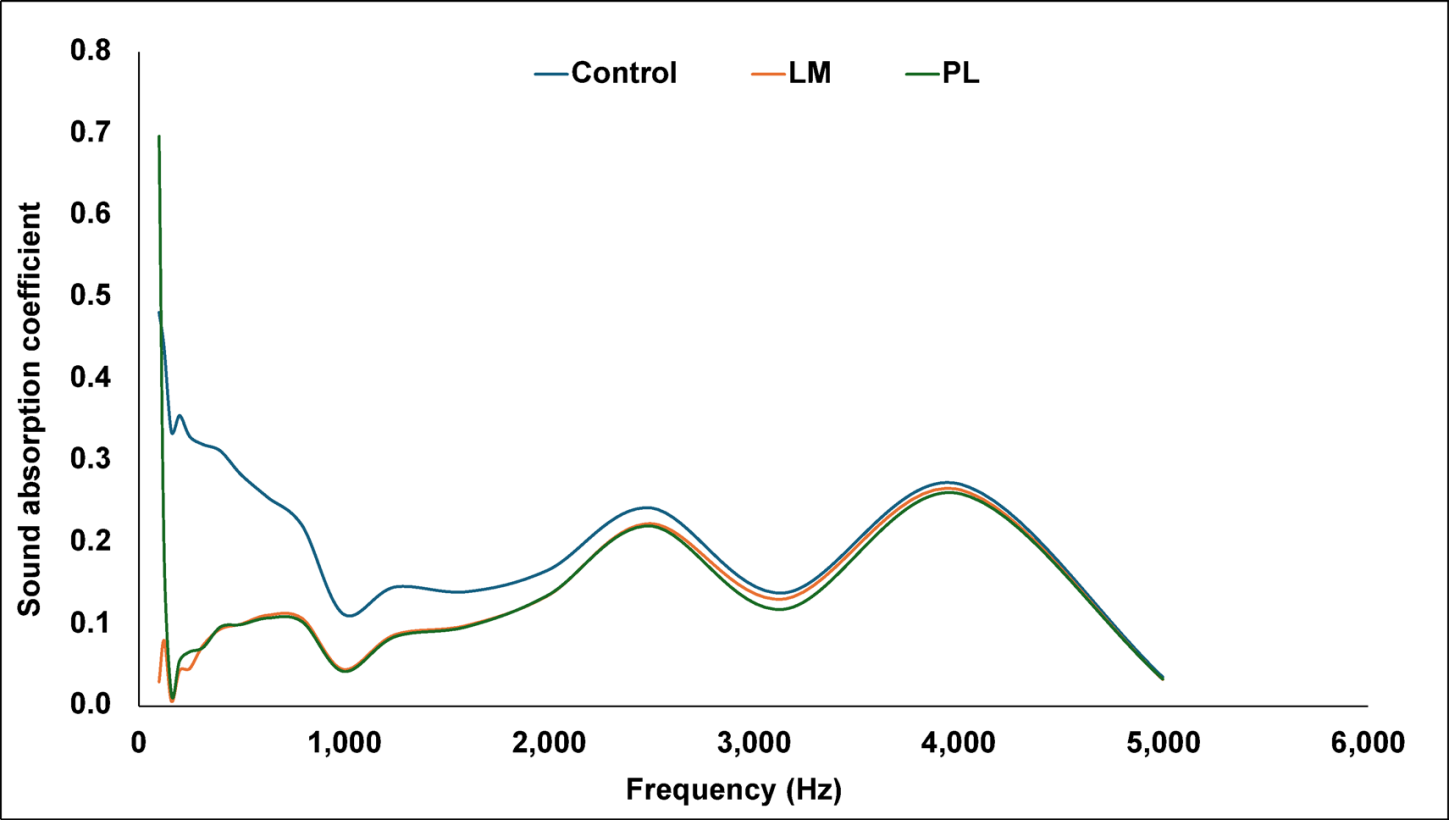
Sound absorption coefficient of the PS composites.

The normal incidence sound absorption property of the composites is presented in Fig. 11. The sound absorption coefficients (ɑ) of LM and PL composites were somewhat similar throughout the absorption spectra with a maximum ɑ value of 0.3. At lower frequencies (150–1000 Hz), there was an observed decrease in sound absorption in the aggregate-filled composites compared to the control samples. This is likely attributed to the packing of the aggregates (as seen in Fig. 3), which decreased the porosity of the composites^49^. However, at higher frequencies, their absorption bands were like that of the control sample. This reflects PS-induced porosity in composites creating voids within the matrix^50^. PL composites had a significantly higher absorption of 0.7 at 100 Hz. This can be attributed to the porosity of the PS fibres and PL voids. Sound waves entering a material encounter friction and viscous losses in the pore network and within the fibre cavities^51^. Overall, the composites had average sound absorption (ɑ < 0.3) in the mid-frequency and high-frequency ranges. In a building application, this could be useful for acoustic panels that can reduce echo and noise. The results further show that adding aggregates to cement composites did not affect the acoustic performance of the composites. The PS itself already introduced a porous fibrous network which makes it superior to dense concrete that has ɑ value of 0.05–0.1^52^.

### Thermal properties

**Fig. 12:**
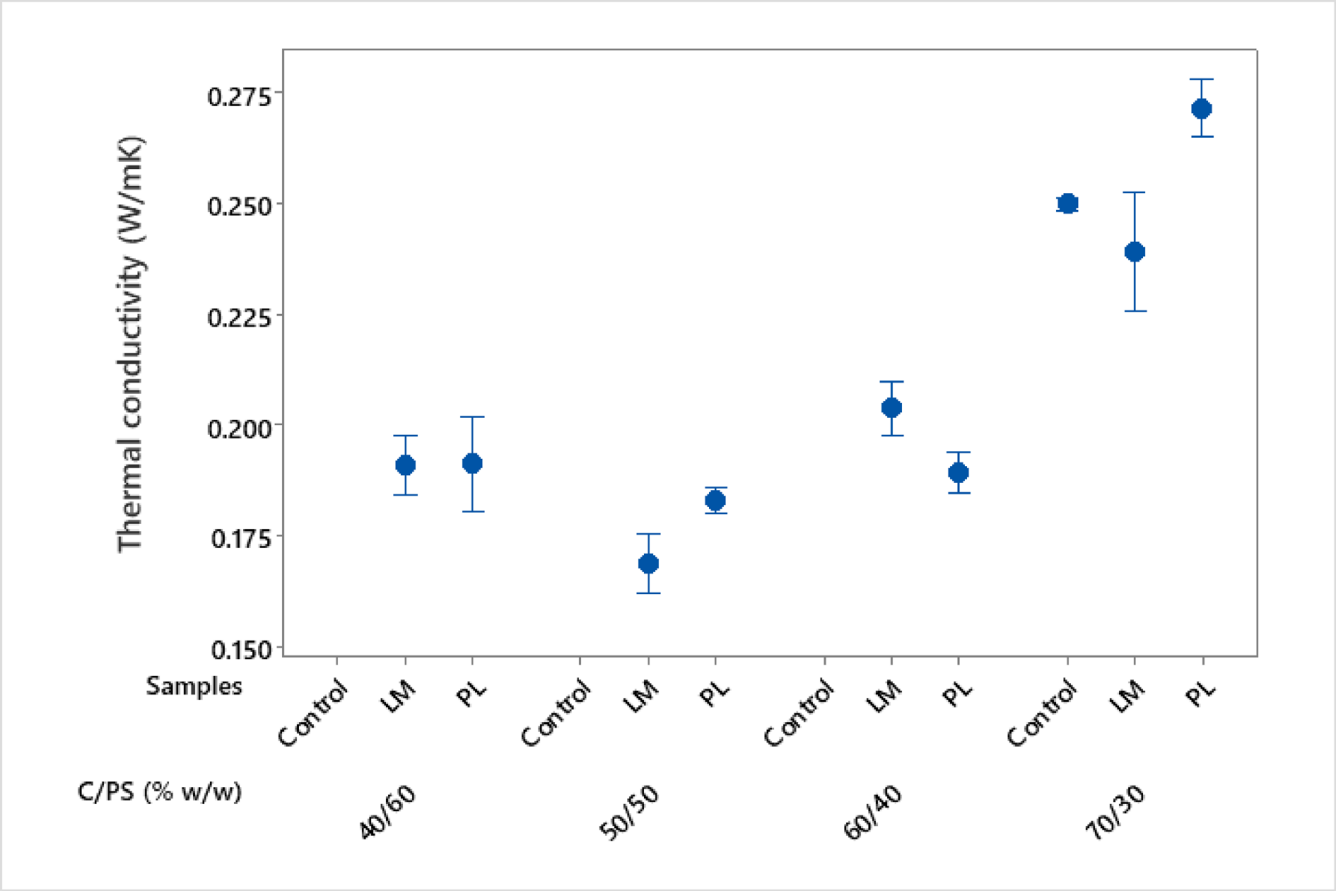
Thermal conductivity of the PS composites.

The average thermal conductivity (λ) of the composite samples is presented in Fig. 12. The thermal conductivity increased with increasing binder content (C/PS ratio). Since hydrated cement yields high density, higher density materials typically have higher thermal conductivity. Cement hydration produces crystalline phases that are more effective at conducting heat than the amorphous materials found in PS^53^. In half of the composite types, PL composites show higher λ values compared to LM. However, PL composites generally show lower thermal conductivity values because the high porosity in PL creates numerous air pockets, enhancing its insulating properties and significantly reducing heat transfer^54^. It is worthy to note that PS fibres can introduce some hygroscopicity, which may increase thermal conductivity if the material is moist^55^. Composites with 7/3 parts (C/PS) showed the highest λ among the composites (0.27 W/mK), whereas those of LM and control were 0.24 and 0.25 W/mK, respectively. These values are within the range reported in other studies for wood cement composites (0.29 W/mK)^2,44^. The high λ in PL could be explained by the overall packing density in the composite compared to that of LM (as seen in Fig. 3). The decrease in λ in LM composites was about 11%. Addition of LM to concrete also decreased thermal conductivity of mortar by up to 15% when 40% of the cement was replaced^23^.

### Fire reaction properties

**Fig. 13:**
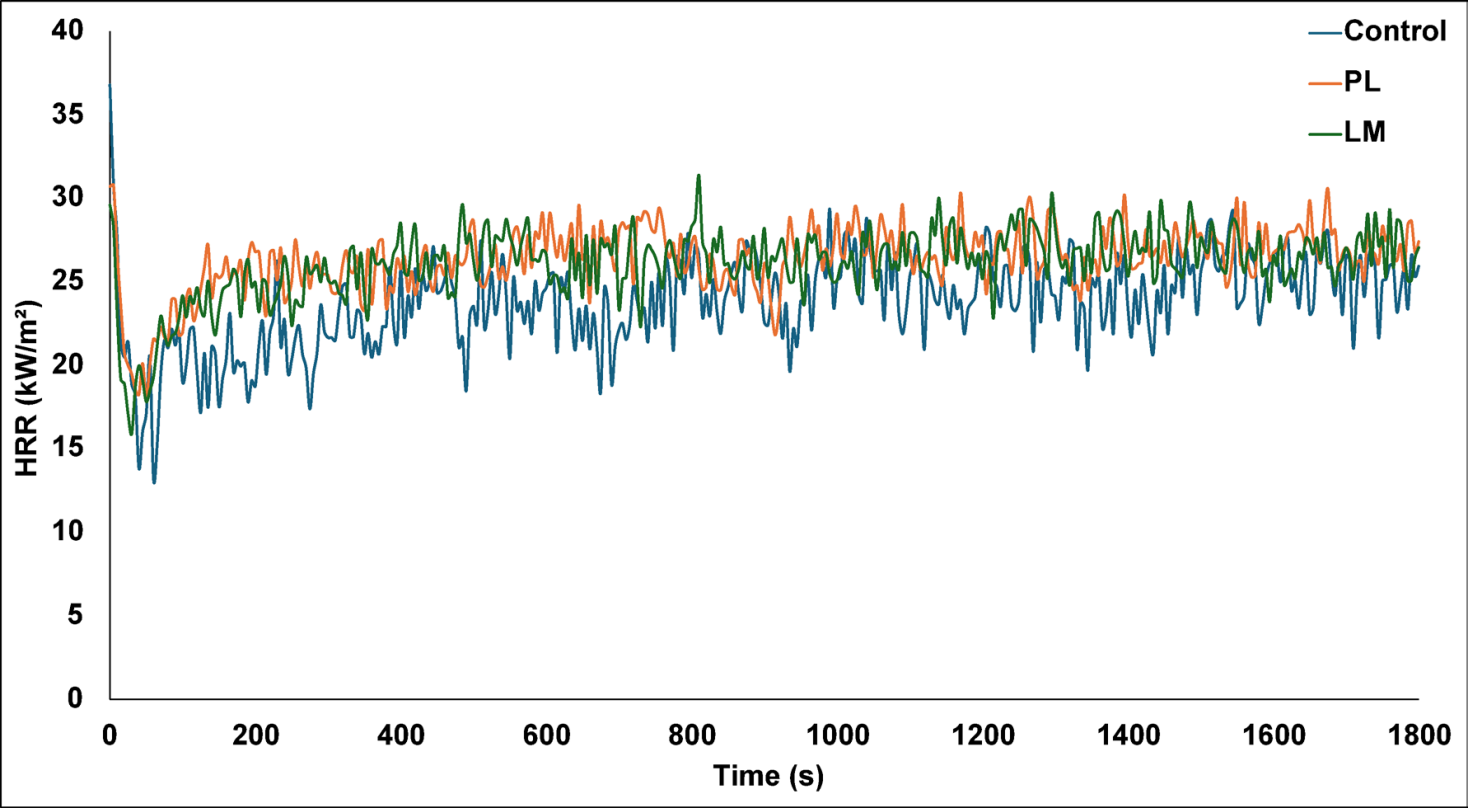
Mean HRR of the PS composites.

The cone calorimetry test showed that the PS composites have good reaction to fire at an irradiance level of 50 kW/m^2^. The mean values of the heat release rate (HRR), total heat release (THR) and mass loss rate (MLR) are presented in Table 4. PL composites exhibited highest mean HRR (26.4 kW/m^2^) compared to LM and the control composites, given at 26.1 and 23.8 kW/m^2^, respectively. Generally, the PS composites had low HRR due to the cement matrix, which acts like a barrier, physically limiting the amount of combustible material exposed at any given time. The total heat released by the composites over 30 min was relatively modest (≤ 23.3 MJ/m^2^), indicating that only a fraction of the composite combusted. The low MLR also suggests that the composites are inherently fire resistant due to the inorganic components. However, Kuqo et al.^56^ observed that low values of HRR and MLR are indicative of the amount of organic content in geopolymer composites. The spectra in Fig. 13 show that although there are small differences in HRR among the PS composite types, control composites had a slightly lower peak compared to LM and PL composites. The aggregates likely acted as microencapsulated phase change materials that can absorb and release thermal energy over time^57^. PL can store thermal energy over longer periods, which can influence HRR^58^. The results also showed that LM composite took a longer time to reach peak HRR. The combination of the heat capacity of CaCO_3_ and its ability to absorb heat through endothermic decomposition can create a heat-sink effect that reduces the effective thermal energy available for the ignition of the LM composite, leading to a longer peak time^59^. While cone calorimetry alone does not assign Euroclass, the low HRR and THR suggests favourable fire reaction performance compared to wood panels.

**Table 4:** Mean HRR, THR and mass loss of composite samples.

Sample type	HRR (kW/m^2^)	THR (MJ/m^2^)	MLR (%, g/s)
Control	23.8	20.6	4.4
PL	26.4	23.3	4.2
LM	26.1	22.9	4.1

### Composite microstructure-property linkages

The packing/porosity, micro-filler effects, and PS fibre bridging collectively explain the observed properties. The density range (732–749 kg/m^3^) and SEM-visible pore network are consistent with the low thermal conductivity (0.17–0.27 W/m·K), as the conductivity in cementitious materials increases with solid fraction and moisture content^60^. The CaCO_3_-rich, ultrafine particles of LM likely contributed nucleation and packing that marginally increased flexural and compressive response relative to PL at equal dosage. This complements earlier reports on LM as a beneficial filler^43,61^. Conversely, the cellular morphology of PL increases entrained porosity, favouring insulation but trading some strength at higher contents^62,63^. DIC maps showed diffuse strain fields and vertical splitting with evidence of fibre-bridging, explaining the post-peak toughness. In fire testing, low HRR/THR reflect the inorganic matrix and low combustible fraction. Lime mud’s delayed peak HRR is compatible with CaCO_3_ endothermy. Overall, these mechanisms reconcile our results with earlier LM/PL literature, supporting the addition of small fractions (5 wt.%) of aggregates for balanced performance.

### Environmental aspects

Substituting cement with PS (up to 50 wt.%) and adding small fractions of LM or PL reduces clinker demand and diverts industrial residues from landfill. Given that Portland cement production dominates concrete’s embodied greenhouse gas emissions^64^, lowering cement content is a mitigation strategy. PL has a relatively low mass-based embodied CO₂ compared to Portland cement^65^. PS and LM are paper industry by-product and using them in mortars valorises waste stream. The material choices selected in this study support reduced embodied impacts alongside end-of-life benefits (e.g., inert mineral content). However, these claims warrant future life cycle assessment (LCA) studies with product-specific environmental declarations.

## Conclusions

This paper evaluated the influence of using lime mud (LM) and expanded perlite (PL) as aggregates for partial replacement of cement in composite materials. The study demonstrated that pulp sludge (PS), a pulp and paper residue, can be effectively used to produce lightweight cement composites, and that small additions of these aggregates can further enhance the composite performance. A 5% replacement of cement by PL or LM gave the best overall mechanical performance. PS fibres provided significant reinforcement, evidenced by improved post-crack ductility and strain distribution. The composites showed low thermal conductivity, substantially better than ordinary cement materials and comparable to other lightweight cement bonded composites. The sound absorption coefficient was within acceptable range for acoustic applications. These dual insulation properties make the material attractive for energy-efficient, noise-reducing building applications. The composites also showed good fire reaction behaviour. The inorganic components likely protected the fibres, resulting in low heat release and structural integrity maintenance under heat. SEM images confirmed good integration of PS fibres and dispersion of fillers. XRD confirmed no disruptive expansions. The presence of CaCO_3_ from PS and LM as well as amorphous silica from PL may even contribute to long-term strength via secondary hydration.

Upcycled PS and cement composites with small PL or LM incorporation presents a promising new class of building materials that combine lightweight, insulating, and load-bearing capabilities. They offer a solution for non-structural elements where reduced weight and improved thermal and acoustic performances are desired, such as prefabricated wall panels, insulating boards, etc. Future work should investigate long-term durability (e.g., freeze–thaw resistance, biodegradation of fibres over years, etc.). However, the results presented in this study support the feasibility of producing these composites on an industrial scale, which could help pulp and paper industries reduce waste and provide the construction sector with more sustainable material options.

Future research should quantify composite durability (e.g., cyclic moisture, freeze–thaw), long-term fibre-matrix stability, and conduct classification-oriented testing such as full reaction-to-fire (Euroclass fire rating). Environmental gains should be substantiated via a cradle-to-gate LCA and sensitivity to PS, LM, or PL sourcing.

## Data Availability

The datasets generated during and/or analysed during the current study are available from the corresponding author on reasonable request.
